# Increased COUP-TFII Expression Mediates the Differentiation Imbalance of Bone Marrow-Derived Mesenchymal Stem Cells in Femoral Head Osteonecrosis

**DOI:** 10.1155/2019/9262430

**Published:** 2019-12-08

**Authors:** Sheng-Hao Wang, Guo-Hau Gou, Chia-Chun Wu, Hsain-Chung Shen, Leou-Chyr Lin, Ru-Yu Pan

**Affiliations:** ^1^Department of Orthopaedics, Tri-Service General Hospital, Taipei, Taiwan; ^2^Graduate Institute of Medical Science, National Defense Medical Center, Taipei, Taiwan

## Abstract

**Objective:**

Bone marrow-derived mesenchymal stem cells (BMSCs) have multilineage differentiation potential, which allows them to progress to osteogenesis, adipogenesis, and chondrogenesis. An imbalance of differentiation between osteogenesis and adipogenesis will result in pathologic conditions inside the bone. This type of imbalance is also one of the pathological findings in osteonecrosis of the femoral head (ONFH). Chicken ovalbumin upstream promoter-transcription factor II (COUP-TFII) was previously reported to mediate the differentiation of mesenchymal stem cells. This study investigated the expression of the osteogenesis regulator Runx2, osteocalcin, the adipogenesis regulator PPAR*γ*, and COUP-TFII in the femoral head tissue harvested from ONFH patients, and characterized the effect of COUP-TFII on the differentiation of primary BMSCs.

**Methods:**

Thirty patients with ONFH were recruited and separated into 3 groups: the trauma-, steroid- and alcohol-induced ONFH groups (10 patients each). Bone specimens were harvested from patients who underwent hip arthroplasty, and another 10 specimens were harvested from femoral neck fracture patients as the control group. Expression of the osteogenesis regulator Runx2, osteocalcin, the adipogenesis regulator PPAR*γ*, C/EBP-α, and COUP-TFII was analyzed by Western blotting. Primary bone marrow mesenchymal cells were harvested from ONFH cells treated with COUP-TFII RNA interference to evaluate the effect of COUP-TFII on MSCs.

**Results:**

ONFH patients had significantly increased expression of the adipogenesis regulator PPAR*γ* and C/EBP-α and decreased expression of the osteogenesis regulator osteocalcin. ONFH bone tissue also revealed higher COUP-TFII expression. Immunohistochemical staining displayed strong COUP-TFII immunoreactivity adjacent to osteonecrotic trabecular bone. Increased COUP-TFII expression in the bone tissue correlated with increased PPAR*γ* and decreased osteocalcin expression. Knockdown of COUP-TFII with siRNA in BMSCs reduced adipogenesis and increased osteogenesis in mesenchymal cells.

**Conclusion:**

Increased COUP-TFII expression mediates the imbalance of BMSC differentiation and progression to ONFH in patients. This study might reveal a new target in the treatment of ONFH.

## 1. Introduction

Femoral head osteonecrosis, also known as avascular necrosis, was previously considered a pathological state that causes decreased vascular supply to the subchondral bone of the femoral head, resulting in osteocyte death and collapse of the articular surface. Multiple factors have been implicated in the development of osteonecrosis of the femoral head (ONFH). In particular, nontraumatic ONFH is directly or indirectly related to disturbance of the vascular supply of the femoral head. Recent data suggest that ONFH is a multisystemic disease rather than a simple disease of the femoral head [1–6]. There are indications that osteonecrosis can be induced by decreased blood supply, increased marrow pressure, abnormal coagulation, or toxic effects on bone cells. Many nontraumatic conditions are associated with osteonecrosis [1, 7–9], including corticosteroid use [10–12], alcohol overuse [4, 13, 14], hemoglobinopathies [15–17], systemic lupus erythematous [18, 19], and coagulation abnormalities [20–22].

Technological advances have enabled studies on the detailed mechanisms of ONFH. An imbalance of bone cell differentiation was noted to be involved in the pathogenesis of ONFH. Abnormal osteogenic differentiation of bone marrow-derived mesenchymal stem cells (BMSCs) [23–25] and enlarged bone marrow fat cells [26] occur in ONFH. Recent clinical studies have shown good results after treatment with statins and low molecular weight heparin [27, 28].

Mesenchymal stem cells have multilineage differentiation potential, which allows them to progress to osteogenesis, adipogenesis, and chondrogenesis. An imbalance between osteogenesis and adipogenesis will result in pathologic conditions inside the bone. Chicken ovalbumin upstream promoter-transcription factor II (COUP-TFII), a member of the orphan nuclear receptor superfamily, was previously noted to be widely expressed in developing organs and to play an important role in cellular growth, differentiation, and organ development [29–31]. In recent studies, high levels of COUP-TFII were associated with the formation of adipose cells from MSCs, and low levels of COUP-TFII increased osteoblast differentiation [32–34]. Ablation of COUP-TFII in mice can increase bone density and suppress fat formation, implying that COUP-TFII may act as an important regulator of osteoblast and adipocyte differentiation [33]. This regulatory mechanism is very similar to the pathological findings in ONFH. We hypothesized that decreased bone density and adipocyte accumulation in osteonecrotic lesions may be linked to COUP-TFII expression.

To clarify the role of COUP-TFII in ONFH, we investigated the expression of the osteogenesis regulator Runx2, osteocalcin, the adipogenesis regulator PPAR*γ*, and COUP-TFII in the femoral head tissue harvested from ONFH patients and evaluated correlations among these factors. Then, we further explored the effect of COUP-TFII on the differentiation of primary BMSCs.

## 2. Materials and Methods

### 2.1. Patients

A total of 30 patients with ONFH were recruited in this study and separated into 3 groups: the trauma-, steroid-, and alcohol-induced groups (each group included 10 patients). The inclusion criteria were patients diagnosed with Ficat stage III ONFH who received joint arthroplasty. The diagnosis and severity were based on plain film and MRI images and then classified by the Ficat system. We excluded patients with malignancy, renal osteodystrophy, metabolic bone diseases, and Paget's disease. The bone specimens were harvested from the center of the resected femoral head for further study. Another 10 healthy patients with acute femoral neck fracture were recruited, and they also all received hip arthroplasty as treatment. Bone specimens from the center of the femoral head were harvested as controls. There were no age-matched control patients because the initial treatment in young patients with femoral neck fractures was reduction and fixation by screws, not hip arthroplasty. Patient demographic data, including age, gender, and comorbidities, were extracted. Comorbidities were assessed using the Deyo-Charlson scoring method from clinical notes [35]. The Institutional Review Board of Tri-service General Hospital approved this study (TSGHIRB No. 2-105-05-058), and every patient signed an informed consent form before the operation.

### 2.2. Histological Analysis of Bone Tissue Samples

All resected femoral head samples were fixed in 10% formalin for 3 days and then decalcified in a 10% EDTA-Tris buffer for 21 days. Finally, the resultant samples were embedded in paraffin, and 5 *μ*m sections of each specimen were prepared. Hematoxylin and eosin (H&E) staining was routinely performed to analyze morphology. The trabecular bone volume in the femoral heads was measured by ImageJ software (Developer Wayne Rasband at National Institutes of Health) according to Dempster et al. [36], and the percentage of trabecular bone volume/total volume (BV/TV) of the femoral heads was calculated.

### 2.3. Western Blot Analysis

Western blotting was performed using proteins isolated from osteonecrotic regions of the femoral heads. Necrotic bone tissues were harvested from the subchondral area (approximately 3~5 mm below the cartilage). The bone tissues were washed twice with phosphate-buffered saline (PBS). Cytosolic protein and nuclear protein were extracted with cell lysis buffer and nuclear lysis reagent with a ProteoJET Cytoplasmic and Nuclear Protein Extraction kit (Fermentas) to which protease inhibitors were added. Cell extracts were microcentrifuged for 5 min at 10,000 g, and the supernatants were collected. Protein extracts were separated on a 10% SDS polyacrylamide gel with electrophoresis for 2.5 h at 80 V in buffer containing 25 mM Tris, 200 mM glycine and 0.1% SDS. After electrophoresis, the proteins were transferred to a polyvinylidene fluoride membrane (Millipore) with a semidry transfer unit at 20 V for 30 min. The membranes were blocked for 1 h with 5% skim milk in Tris-buffered saline (TBS) (20 mM Tris base pH 7.6, 150 mM NaCl) containing 1% Tween-20 at room temperature and incubated for 1 h at room temperature or overnight at 4°C with the GeneTex COUP-TFII beta-actin primary antibodies. The membranes were then washed three times for 10;min with TBS containing 1% Tween-20. After washing was complete, the membrane was incubated for 1 h at room temperature with appropriate secondary antibodies conjugated to horseradish peroxidase (HRP) and washed three times for 10 min with TBS containing 1% Tween-20. Bound antibody was detected using Immobilon Western Chemiluminescent HRP Substrate (Millipore).

### 2.4. Immunohistochemistry

The paraffin sections were incubated with avidin, biotin, and bovine serum albumin to avoid nonspecific staining. The sections were then incubated with an anti-COUP-TFII antibody (1:200; ab64849, Abcam) dissolved in 1% BSA (BSA)/PBS (PBS) overnight at 4°C. After washing, the sections were stained with anti-mouse IgG (1:200; ab6788, Abcam) for 30 min. Finally, 3,3′-diaminobenzidine (Sigma-Aldrich; Merck KGaA) was used to detect the bound antibody, and the sections were counterstained with hematoxylin.

### 2.5. Primary BMSCs

Under sterile conditions, primary BMSCs were obtained from bone marrow (BM) cells from spongy bone collected from the femoral head of osteonecrotic patients using a modified protocol [37] originally described by Haynesworth et al. [38]. Trabecular bone fragments were harvested from the resected femoral head using a bone curette, transferred to conical tubes containing DMEM (Gibco), and ground extensively with surgical scissors and a pestle. Then, the bone fragments were washed repeatedly with DMEM, diluted with phosphate-buffered saline, added to Ficoll extraction liquid and centrifuged (1000 rpm) for 5 min. The pellet containing bone plugs and released cells was resuspended in high D-Glucose (4.5 g/L) DMEM containing 10% fetal calf serum (FBS, HyClone) and 1% EDTA, plated at a density of 10 × 10^6^ cells per 100 cm^2^ tissue culture flask and maintained at 37°C in a 5% CO_2_ incubator for 2 h. The medium was changed every 48 h, and nonadherent cells were collected and removed until the cell cultures reached confluency.

### 2.6. Identification of Human BMSCs

BMSCs were characterized using flow cytometry (FACSCalibur). The P4 BMSCs were digested with 0.25% trypsin/EDTA and then incubated with DMEM-LG to terminate the digestion. The solution was centrifuged for 5 min, and the cells were washed and resuspended with a BD Human MSC Analysis Kit and cold PBS. Human positive marker CD105 PerCP-Cy5.5, CD73 APC, CD90 FITC and negative marker cocktail CD45/CD34/CD11b/CD19/HLA-DR PE were added to the cell suspension. The cells were washed twice in PBS/4% FCS and resuspended in PBS/4% FCS with 1 *µ*g/ml propidium iodide (PI). The samples were analyzed with Cell Quest software (Beckman Coulter).

### 2.7. Osteogenic and Adipogenic Differentiation of Human BMSCs

For osteogenic and adipogenic differentiation, cells in standard medium (osteogenic differentiation) or DMEM with 10% FBS (adipogenic differentiation) were plated in 6-well plates or chamber slides and grown to confluence. At confluence, osteogenic differentiation was induced with the standard osteogenic medium composed of *α*-MEM 10% FCS supplemented with 50 *μ*g/ml ascorbic acid and 10 mM *β*-glycerophosphate, whereas adipogenic differentiation was induced with adipose induction medium containing 10 *µ*g/ml insulin, 1 *µ*M dexamethasone, 0.5 mM 3-isobutyl-1-methylxanthine, and 100 *µ*M indomethacin. The medium was changed every 2 days for up to 2 weeks for adipogenic stimulation and up to 4 weeks for osteogenic stimulation.

### 2.8. RNA Interference Transfection

Cells were transiently transfected with specific siRNAs to knock down COUP-TFII. RNA interference sequences were submitted to NCBI blast, aligned with the COUP-TFII mRNA and obtained from the National RNAi Core Facility Academia Sinica in Taiwan. The COUP-TFII siRNA sequences were GCUUUGGAAGAAUACGUUAtt (sense) and UAACGUAUUCUUCCAAAGCac (antisense). Primary bone marrow-derived stem cells were transiently transfected with 1 *μ*g of plasmids and scrambled controls using Lipofectamine 2000 (Invitrogen). Then, the cells were treated with and without COUP-TFII siRNA for 72 h.

### 2.9. Oil Red O Staining

Following adipose induction, the cells were gently washed twice with PBS. Subsequently, the cells were fixed with 4% paraformaldehyde for 10 min and washed twice with deionized water. Oil Red O solution (Sigma) was added for 20 min and subsequently washed twice with PBS.

### 2.10. Alizarin Red Staining

Following osteoblast induction, the medium was removed, and the cells were washed and fixed with 70% ethanol for 1 h and then washed twice with PBS. A 40 mM AR-S solution was added to 2% ethanol for 3 min, and the cells were then washed and photographed.

### 2.11. Statistical Analysis

Statistical analysis was performed using IBM SPSS statistical software (version 20.0). Statistical analysis was carried out by Student's *t* test. All of the results are presented as the mean ± standard deviation. *P* < 0.05 was considered to indicate a statistically significant difference. All of the results are presented as the mean ± standard deviation.

## 3. Results

### 3.1. Comparison of Differences in Age, Gender, and Comorbidity between the ONFH Group and the Control Group

This study investigated whether decreases in osteoblasts, increases in adipocytes, and altered COUP-TFII expression in bone specimens were linked to the occurrence of ONFH. The 30 patients in the ONFH groups were all diagnosed with Ficat stage III osteonecrosis under the evaluation of X-ray and MRI. In the steroid-induced ONFH group, there were 10 ONFH patients (6 females and 4 males, average 42.3 ± 6.5 years); in the alcohol-induced ONFH group, 10 ONFH patients (4 females and 6 males, average 50.3 ± 5.5 years) were recruited; and in the traumatic ONFH group, 10 ONFH patients (4 females and 6 males, average 52.7 ± 11.3 years) were recruited. In the control group (femoral neck fracture), 10 patients (5 females and 5 males, average 77.9 ± 6.6 years) were recruited. The ages of ONFH patients in the steroid-induced ONFH group, alcohol-induced ONFH group, and traumatic ONFH group were all significantly lower than the age of patients in the control group (*P* < 0.001). There were no significant differences in gender or comorbidities between these ONFH groups and the control group (Table 1).

### 3.2. Increased Expression of Adipogenesis-Related Factors and Decreased Expression of Osteogenesis-Related Factors Were Noted in the ONFH Group

**Figure 1:**
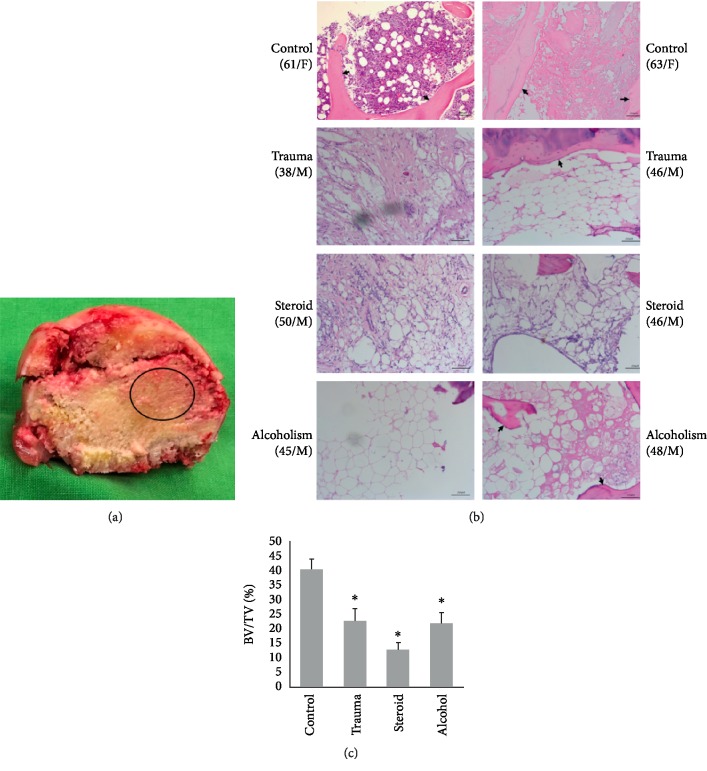
(a) The femoral head was extracted and split in half, and the necrotic bone around the center of the femoral head (circle) was harvested. (b) Compared with the control group, the trauma, steroid, and alcohol groups all showed increased numbers of adipocytes and decreased trabecular bone (black arrows) in the H&E sections (100x). (c) The BV/TV of femoral heads in the osteonecrotic groups all showed significant decreases compared with those in the control group. (Data are presented as mean ± SD; *t *test, ^∗^*P* < 0.05, compared with the control group; BV/TV, bone volume/total volume.)

**Figure 2:**
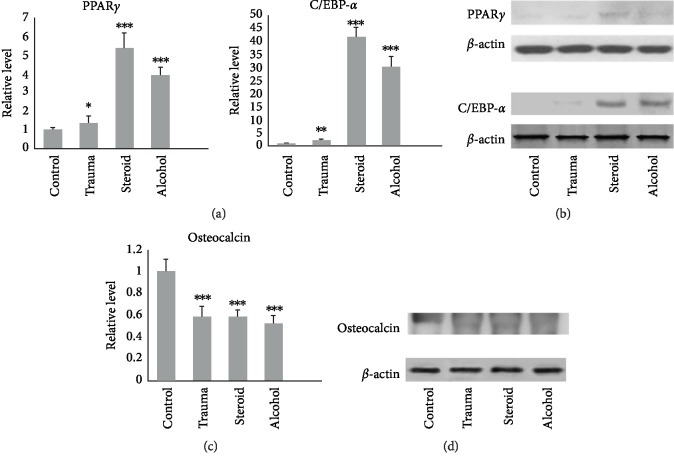
(a and b) In the osteonecrotic groups, the key factors (PPAR*γ* and C/EBP-*α*) controlling adipogenesis were significantly decreased compared with the control group. (c and d) On the other hand, the osteogenesis marker (osteocalcin) was significantly decreased in all of the osteonecrotic groups. (Data are presented as mean ± SD; *t *test, ^∗^*P* < 0.05, ^∗∗^*P* < 0.01, ^∗∗∗^*P* < 0.001, and the difference was statistically significant compared with the control group.)

Bone specimens were harvested from the area surrounding the center of the femoral head for further analysis. Histopathological examination revealed reduced trabecular bone and an increased number of adipocytes in H&E-stained sections from the osteonecrotic groups compared with the control samples (Figures 1(a) and 1(b)). In addition to their increased number, the adipocytes in the bone marrow were also increased in size, resulting in reduced space for hematopoietic cells. The quantitative analysis of BV/TV in femoral heads demonstrated significant differences between the control and ONFH groups (*P* < 0.05; Figure 1(c)). Western blot analysis showed that ONFH patients in the traumatic ONFH group (*P* = 0.045), steroid-induced ONFH group (*P* < 0.001), and alcohol-induced ONFH group (*P* < 0.001) had significantly higher PPAR*γ* protein expression than those in the femoral neck fracture group (Figures 2(a) and 2(b)). Significantly higher C/EBP-*α* expression was also noted in samples from ONFH patients than in those from controls (*P* = 0.01 in the traumatic ONFH group, *P* < 0.001 in the steroid-induced ONFH and alcohol-induced ONFH groups). The ONFH patients had significantly decreased osteocalcin protein expression (*P* < 0.001 in the traumatic, steroid-, and alcohol-induced ONFH groups) compared with those in the femoral neck fracture group (Figures 2(c) and 2(d)).

### 3.3. Increased COUP-TFII Expression in the ONFH Group

**Figure 3:**
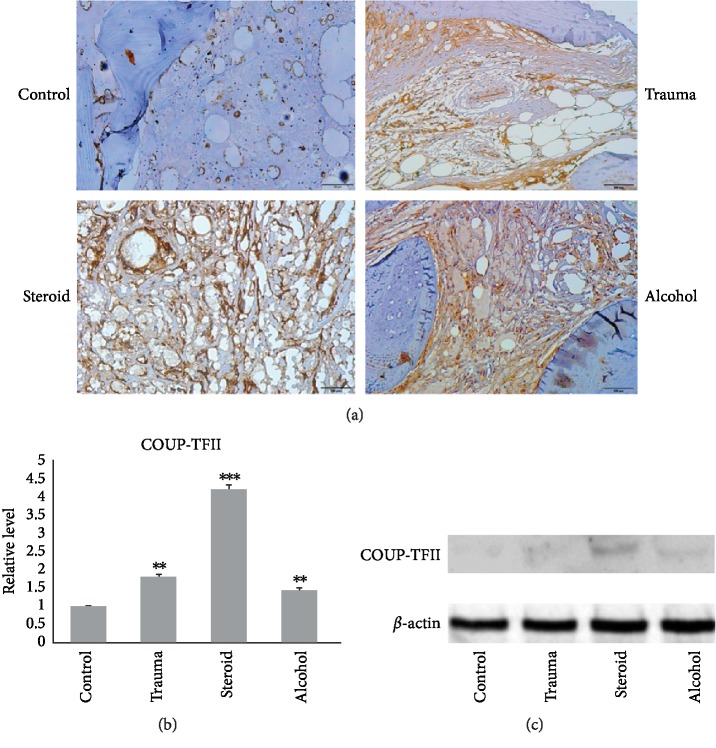
(a) Immunohistochemical images (200x) of osteonecrotic tissue showing COUP-TFII expression in the bone specimens. In the osteonecrotic groups, osteonecrotic lesions displayed strong COUP-TFII expression compared with those in the control group. (b and c) Western blot analysis showed increased expression of COUP-TFII in all the osteonecrotic groups compared with the control group. (Data are means ± SD; *t *test, ^∗^*P* < 0.05, ^∗∗^*P* < 0.05, ^∗∗∗^*P* < 0.001, and the difference was statistically significant compared with the control group.)

Histological analysis was used to compare COUP-TFII expression in bone specimens from ONFH patients with that in bone specimens from the controlled group. In the ONFH groups, the increased number of adipocytes adjacent to the bone tissue exhibited evident COUP-TFII immunoreactivity (brown immunostaining around the cells and in the cytoplasm). However, osteoblasts displayed weak COUP-TFII expression in the control group (Figure 3(a)). Western blot results showed that ONFH patients in the traumatic ONFH group (*P*=0.006), steroid-induced ONFH group (*P* < 0.001) and alcohol-induced ONFH group (*P* = 0.015) had significantly higher COUP-TFII expression levels (Figures 3(b) and 3(c)) than patients in the control group.

### 3.4. COUP-TFII Mediated Glucocorticoid-Induced Adipogenesis in Cultured Primary BMSCs

**Figure 4:**
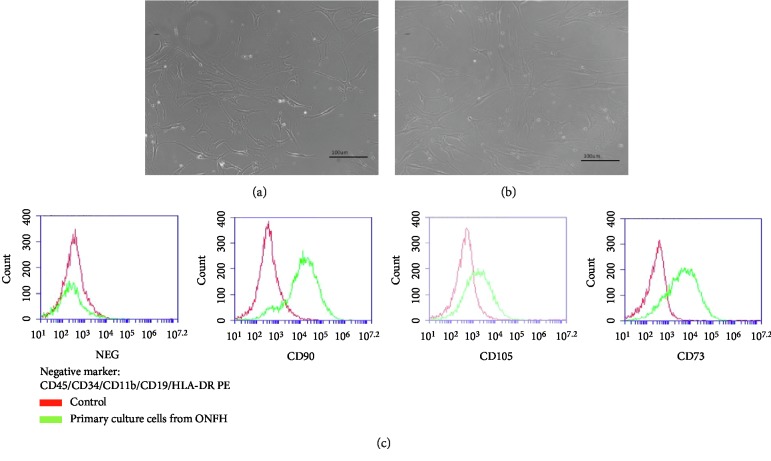
Morphological analyses of bone marrow mesenchymal stem cells (BMSCs). (a) Initial primary BMSCs from osteonecrotic patients demonstrated spindle-shaped morphology (magnification, 200x). (b) Following four generations (16 days), the cells presented with a similar spindle morphology (magnification, 200x). (c) Flow cytometry results of mesenchymal stem cells (MSC) showed that the cells were positive for the markers CD90, CD105, and CD73 and negative for CD45/CD34/CD11b/CD19/HLA-DR PE.

This study evaluated whether COUP-TFII controlled adipogenesis of primary BMSCs from ONFH patients. To study the potential role of COUP-TFII in mesenchymal cell differentiation, primary BMSCs harvested from ONFH tissues were harvested and cultured in culture medium and were capable of being passaged several times. Primary cultured BMSCs exhibited spindle-shaped morphology. After passaging four times, the cells had a similar long spindle shape (Figures 4(a) and 4(b)). When the results were evaluated with flow cytometry, these BMSCs were found to express the positive surface markers CD90, CD105, and CD73 and the negative surface markers CD45/CD34/CD11b/CD19/HLA-DR PE (Figures 4(c) and [Supplementary-material supplementary-material-1]). These results confirmed that these isolated cells were BMSCs.

**Figure 5:**
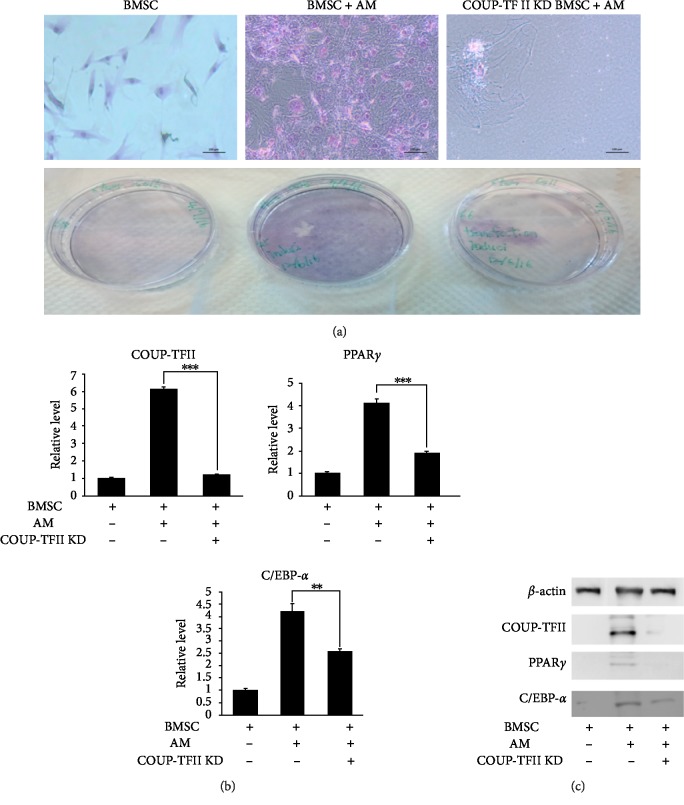
(a) COUP-TFII promotes the adipogenic differentiation of mesenchymal cells. COUP-TFII siRNA-treated BMSC cells and controlled BMSC cells underwent adipocyte differentiation, and cells were stained with oil red O and photographed on day 14. Decreasing adipogenesis was noted after COUP-TFII knockdown (magnification, 200x). (b and c) Impaired adipogenesis in COUP-TFII-depleted cells was confirmed by western blotting, with decreased expression of PPAR*γ* and C/EBP-*α*. (AM: adipogenic medium; KD: knockdown; Data are presented as mean ± SD; *t *test, ^∗∗^*P* < 0.01, ^∗∗∗^*P* < 0.001.)

Then, the effect of COUP-TFII on the adipogenesis of primary BMSCs was evaluated by transfection with COUP-TFII RNA interference. After COUP-TFII RNA interference, the level of COUP-TFII was significantly diminished (Figures 5(b) and 5(c)). Oil red O staining showed that the influenced COUP-TFII expression attenuated adipogenesis and decreased the number of adipocytes in the adipogenesis culture medium compared with the control tissue samples (Figure 5(a)). In the Western blot analysis, COUP-TFII RNA interference significantly diminished COUP-TFII expression, which further decreased C/EBP*α* and PPAR*γ* expression (Figures 5(b) and 5(c)).

### 3.5. Knockdown of COUP-TFII Increased the Osteogenesis of Primary BMSCs from the ONFH Group

**Figure 6:**
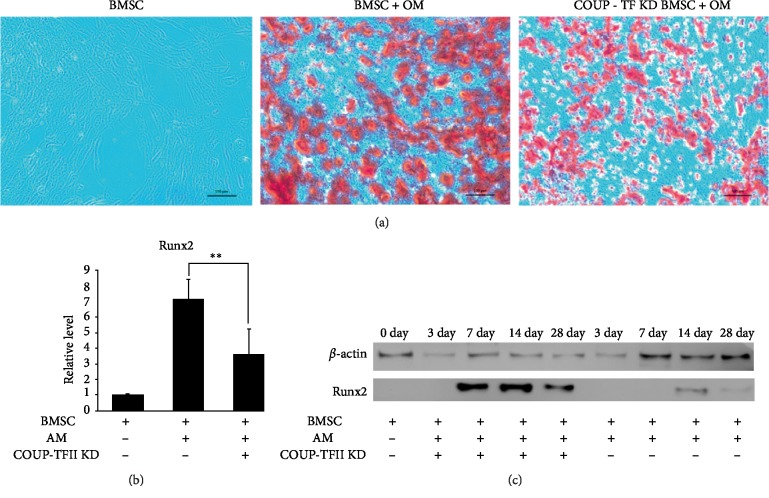
(a) Alizarin red staining of BMSCs that underwent osteogenesis for 28 days. When cultured in osteogenic medium, COUP-TFII knockdown BMSCs showed increased osteogenic differentiation compared with control BMSCs (magnification, 200x). (b and c) Increased osteogenesis in COUP-TFII-depleted cells was confirmed by western blotting with increased expression of Runx2. (OM: osteogenic medium; KD: knockdown; data are presented as the means ± SD; *t *test, ^∗∗^*P* < 0.01.)

Next, the function of COUP-TFII in the osteogenesis of primary BMSCs was evaluated. COUP-TFII RNA interference was used to transiently block COUP-TFII expression in BMSCs under osteogenic stimulation. Alizarin red staining was used to examine increased bone nodule formation in COUP-TFII knockdown BMSCs (Figure 6(a)), and Western blot analysis showed a significant increase in key osteogenic transcriptional factors (Runx2) even up to 28 days after osteogenic stimulation (Figures 6(b) and 6(c)). Collectively, our data demonstrate the critical role of COUP-TFII as a positive regulator of adipogenesis and a negative regulator of osteogenesis in progenitor cells.

## 4. Discussion

The main finding of the study is that increased COUP-TFII expression mediates an imbalance of BMSC differentiation and progression to ONFH in patients. COUP-TFII has been previously reported to play an important role in embryogenesis and is widely detected in developing organs [39–41]. It is also involved in the regulation of mesenchymal cell commitment and differentiation through adipogenesis, osteogenesis, or chondrogenesis [33, 41]. The imbalance of BMSC differentiation is similar to the pathological processes that occur in the osteonecrotic femoral head tissue. At present, no research has been performed to link COUP-TFII expression to the incidence of ONFH. In this study, COUP-TFII was verified to act as an important promoting factor that increases adipogenesis and inhibits osteogenesis, which deteriorates the inner structure of the femoral head and ultimately results in the collapse of subchondral bone in ONFH.

Good function of bony homeostasis relies on the balance of differentiation between osteogenesis and adipogenesis to maintain a well-constructed bony structure of the femoral head. Although the pathological incidence of increased adipogenesis and decreased osteogenesis has been revealed in ONFH tissues, the crucial molecules that potentially mediate these changes in MSC transformation in ONFH lesions remain unknown. This study revealed that increased induction of adipogenesis and decreased osteogenesis were the prominent reactions in ONFH lesions, as determined by the distinct finding of increased PPAR*γ* expression with intense adipogenesis and decreased RUNX2 expression with diminished osteogenesis in these osteonecrotic tissues.

MSCs are pluripotent progenitors that are present in the stromal fraction of many adult tissues. They are capable of undergoing differentiation into adipocytes, osteoblasts, and chondrocytes in response to specific stimuli [42, 43]. In bone tissue, osteogenesis is programmed for making strong skeletal structures and resisting external pressure. Many diseases, including osteoporosis and osteonecrosis, have been shown to be significantly associated with imbalances between osteogenic and adipogenic differentiation of BMSCs [44, 45]. Recent studies also revealed that ONFH is mainly caused by increased differentiation of BMSCs into adipocytes and decreased bone osteogenesis [46, 47]. This study showed increased adipogenesis and decreased osteogenesis in necrotic regions of the femoral head. Increased PPAR*γ* and C/EBP-*α* expression were correlated with the promotion of cell adipogenesis. Decreased Runx2 and osteocalcin were correlated with decreased osteogenesis in the osteonecrotic tissue. Immunohistochemical and Western blot analysis showed that bone cells in the osteonecrotic tissue expressed strong COUP-TFII activity. These findings suggest that COUP-TFII could be a potent molecule that regulates the differentiation of mesenchymal stem cells in osteonecrotic lesions. We further evaluated the effect of COUP-TFII by COUP-TFII RNA interference, which also regulated both PPAR*γ* and RUNX2 expression resulting in increased adipogenesis and decreased osteogenesis of primary BMSCs in ONFH. Altered COUP-TFII expression in ONFH reveals a new molecular target associated with the pathological progression in ONFH. This study is the first to evaluate the correlation of COUP-TFII expression with the occurrence of ONFH.

At present, it is still unknown what pathological mechanism upregulates COUP-TFII expression in ONFH patients. In this study, excessive corticosteroid usage, alcohol consumption, and trauma-induced ONFH all increased COUP-TFII expression. The primary cell culture examination also showed that knockdown of COUP-TFII decreased dexamethasone-induced adipogenesis and increased osteogenesis in primary BMSCs. This study revealed that COUP-TFII in bone cell actively responds to glucocorticoid-, alcohol-, and trauma-induced stress. This COUP-TFII-mediated imbalance in mesenchymal cell differentiation provides novel evidence in ONFH.

There are some limitations of this study. The ages of the patients in the osteonecrosis groups and in the controlled group differed significantly because internal fixation is the primary surgical indication for acute femoral neck fractures in young patients. However, previous studies have revealed an inverse relationship between osteogenesis and adipogenesis in the development of osteoporosis in patients with natural aging compared with young people [48–50]. The exact molecular mechanisms involved in the promotion of COUP-TFII expression in ONFH remain unclear. Many factors might directly or indirectly affect the expression of COUP-TFII. Increased COUP-TFII expression is correlated with corticosteroid use, excessive alcohol consumption, posttraumatic changes in the femoral head, and the associated increased adipogenesis and attenuated osteogenesis in osteonecrotic lesions. Further animal studies might be required to verify the effect of this factor as a therapeutic target for ONFH.

## Figures and Tables

**Table 1:** Patient demographics.

Variables	Control	Trauma		Steroid		Alcohol	
	*n* = 10	*n* = 10	*P* value	*n* = 10	*P* value	*n* = 10	*P* value
Age (years)	77.9 ± 6.6	52.7 ± 11.3	<0.001	42.3 ± 6.5	<0.001	50.3 ± 5.5	<0.001
Gender			0.337		0.337		0.337
Male	5	6		4		6	
Female	5	4		6		4	
CCI^∗^	0.88 ± 0.69	0.63 ± 0.52	0.157	0.82 ± 0.60	0.399	0.70 ± 0.68	0.255

^∗^CCI: Charlson *comorbidity* index (nonage adjusted).

## Data Availability

The data used to support the findings of this study are available from the corresponding author upon request.

## Additional Points

Disclaimer. The author, their immediate family, and any research foundation with which they are affiliated have not received any financial payments, other benefits or writing assistance from any commercial entity related to the subject of this article.

## Supplementary Materials

Supplementary Materials Flow cytometric analysis of bone marrow mesenchymal stem cells (BMSCs). After culturing for 16 days, the majority of bone marrow-derived stem cells express the markers CD90, CD105, and CD73, which are characteristic of mesenchymal stem cells, and are negative for CD45/CD34/CD11b/CD19/HLA-DR PE.

## References

[1] 
Arlet J. (1992). Nontraumatic avascular necrosis of the femoral head. past, present, and future. *Clinical Orthopaedics and Related Research*.

[2] Mankin H. J. (1992). Nontraumatic necrosis of bone (osteonecrosis). *The New England Journal of Medicine*.

[3] Mont M. A., Hungerford D. S. (1995). Non-traumatic avascular necrosis of the femoral head. *The Journal of Bone and Joint Surgery American Volume*.

[4] Lavernia C. J., Sierra R. J., Grieco F. R. (1999). Osteonecrosis of the femoral head. *The Journal of the American Academy of Orthopaedic Surgeons*.

[5] Malizos K. N., Karantanas A. H., Varitimidis S. E., Dailiana Z. H., Bargiotas K., Maris T. (2007). Osteonecrosis of the femoral head: etiology, imaging and treatment. *European Journal of Radiology*.

[6] Larson A. N., McIntosh A. L., Trousdale R. T., Lewallen D. G. (2010). Avascular necrosis most common indication for hip arthroplasty in patients with slipped capital femoral epiphysis. *Journal of Pediatric Orthopedics*.

[7] Jacobs B. (1978). Epidemiology of traumatic and nontraumatic osteonecrosis. *Clinical Orthopaedics and Related Research*.

[8] Hungerford D. S., Lennox D. W. (1985). The importance of increased intraosseous pressure in the development of osteonecrosis of the femoral head: implications for treatment. *The Orthopedic Clinics of North America*.

[9] Etienne G., Mont M. A., Ragland P. S. (2004). The diagnosis and treatment of nontraumatic osteonecrosis of the femoral head. *Instructional Course Lectures*.

[10] Zaidi M., Sun L., Robinson L. J. (2010). ACTH protects against glucocorticoid-induced osteonecrosis of bone. *Proceedings of the National Academy of Sciences of the United States of America*.

[11] Sakaguchi M., Tanaka T., Fukushima W., Kubo T., Hirota Y. (2010). Impact of oral corticosteroid use for idiopathic osteonecrosis of the femoral head: a nationwide multicenter case-control study in Japan. *Journal of Orthopaedic Science : Official Journal of the Japanese Orthopaedic Association*.

[12] Li X., Jin L., Cui Q., Wang G. J., Balian G. (2005). Steroid effects on osteogenesis through mesenchymal cell gene expression. *Osteoporosis International: A Journal Established as Result of Cooperation between the European Foundation for Osteoporosis and the National Osteoporosis Foundation of the USA*.

[13] Wang Y., Li Y., Mao K., Li J., Cui Q., Wang G. J. (2003). Alcohol-induced adipogenesis in bone and marrow: a possible mechanism for osteonecrosis. *Clinical Orthopaedics and Related Research*.

[14] Cui Q., Wang Y., Saleh K. J., Wang G. J., Balian G. (2006). Alcohol-induced adipogenesis in a cloned bone-marrow stem cell. *The Journal of Bone and Joint Surgery American Volume*.

[15] Mukisi M. M., Bashoun K., Burny F. (2009). Sickle-cell hip necrosis and intraosseous pressure. *Orthopaedics & Traumatology, Surgery & Research*.

[16] Buck J., Davies S. C. (2005). Surgery in sickle cell disease. *Hematology/Oncology Clinics of North America*.

[17] Akinyoola A. L., Adediran I. A., Asaleye C. M., Bolarinwa A. R. (2009). Risk factors for osteonecrosis of the femoral head in patients with sickle cell disease. *International Orthopaedics*.

[18] Abu-Shakra M., Buskila D., Shoenfeld Y. (2003). Osteonecrosis in patients with SLE. *Clinical Reviews in Allergy & Immunology*.

[19] Abeles M., Abeles A. M., Urman J., Rothfield N. (2012). Osteonecrosis in SLE. *Rheumatology International*.

[20] Mont M. A., Cherian J. J., Sierra R. J., Jones L. C., Lieberman J. R. (2015). Nontraumatic osteonecrosis of the femoral head: where do we stand today?. *The Journal of Bone and Joint Surgery American Volume*.

[21] Orth P., Anagnostakos K. (2013). Coagulation abnormalities in osteonecrosis and bone marrow edema syndrome. *Orthopedics*.

[22] Garcia F. L., Ramalli E. L., Picado C. H. (2013). Coagulation disorders in patients with femoral head osteonecrosis. *Acta Ortopedica Brasileira*.

[23] Suh K. T., Kim S. W., Roh H. L., Youn M. S., Jung J. S. (2005). Decreased osteogenic differentiation of mesenchymal stem cells in alcohol-induced osteonecrosis. *Clinical Orthopaedics and Related Research.*.

[24] Lee J. S., Lee J. S., Roh H. L., Kim C. H., Jung J. S., Suh K. T. (2006). Alterations in the differentiation ability of mesenchymal stem cells in patients with nontraumatic osteonecrosis of the femoral head: comparative analysis according to the risk factor. *Journal of Orthopaedic Research:Official Publication of the Orthopaedic Research Society*.

[25] Gangji V., Hauzeur J. P., Schoutens A., Hinsenkamp M., Appelboom T., Egrise D. (2003). Abnormalities in the replicative capacity of osteoblastic cells in the proximal femur of patients with osteonecrosis of the femoral head. *The Journal of Rheumatology*.

[26] Motomura G., Yamamoto T., Miyanishi K., Yamashita A., Sueishi K., Iwamoto Y. (2005). Bone marrow fat-cell enlargement in early steroid-induced osteonecrosis—a histomorphometric study of autopsy cases. *Pathology - Research and Practice*.

[27] Pritchett J. W. (2001). Statin therapy decreases the risk of osteonecrosis in patients receiving steroids. *Clinical Orthopaedics and Related Research*.

[28] Kim T. H., Hong J. M., Oh B. (2008). Genetic association study of polymorphisms in the catalase gene with the risk of osteonecrosis of the femoral head in the Korean population. *Osteoarthritis and Cartilage*.

[29] Petit F. G., Jamin S. P., Kurihara I. (2007). Deletion of the orphan nuclear receptor COUP-TFII in uterus leads to placental deficiency. *Proceedings of the National Academy of Sciences of the United States of America*.

[30] Lee C. T., Li L., Takamoto N. (2004). The nuclear orphan receptor COUP-TFII is required for limb and skeletal muscle development. *Molecular and Cellular Biology*.

[31] Kurihara I., Lee D. K., Petit F. G. (2007). COUP-TFII mediates progesterone regulation of uterine implantation by controlling ER activity. *PLoS Genetics*.

[32] Okamura M., Kudo H., Wakabayashi K. (2009). COUP-TFII acts downstream of Wnt/beta-catenin signal to silence PPARgamma gene expression and repress adipogenesis. *Proceedings of the National Academy of Sciences of the United States of America*.

[33] Xie X., Qin J., Lin S. H., Tsai S. Y., Tsai M. J. (2011). Nuclear receptor chicken ovalbumin upstream promoter-transcription factor II (COUP-TFII) modulates mesenchymal cell commitment and differentiation. *Proceedings of the National Academy of Sciences of the United States of America*.

[34] Jeong B. C., Kang I. H., Hwang Y. C., Kim S. H., Koh J. T. (2014). MicroRNA-194 reciprocally stimulates osteogenesis and inhibits adipogenesis via regulating COUP-TFII expression. *Cell Death & Disease*.

[35] Deyo R. A., Cherkin D. C., Ciol M. A. (1992). Adapting a clinical comorbidity index for use with ICD-9-CM administrative databases. *Journal of Clinical Epidemiology*.

[36] Dempster D. W., Compston J. E., Drezner M. K. (2013). Standardized nomenclature, symbols, and units for bone histomorphometry: a 2012 update of the report of the ASBMR Histomorphometry Nomenclature Committee. *Journal of Bone and Mineral Research*.

[37] Noth U., Osyczka A. M., Tuli R., Hickok N. J., Danielson K. G., Tuan R. S. (2002). Multilineage mesenchymal differentiation potential of human trabecular bone-derived cells. *Journal of Orthopaedic Research*.

[38] Haynesworth S. E., Goshima J., Goldberg V. M., Caplan A. I. (1992). Characterization of cells with osteogenic potential from human marrow. *Bone*.

[39] Pereira F. A., Qiu Y., Zhou G., Tsai M. J., Tsai S. Y. (1999). The orphan nuclear receptor COUP-TFII is required for angiogenesis and heart development. *Genes & Development*.

[40] You L. R., Lin F. J., Lee C. T., DeMayo F. J., Tsai M. J., Tsai S. Y. (2005). Suppression of notch signalling by the COUP-TFII transcription factor regulates vein identity. *Nature*.

[41] Li L., Xie X., Qin J. (2009). The nuclear orphan receptor COUP-TFII plays an essential role in adipogenesis, glucose homeostasis, and energy metabolism. *Cell Metabolism*.

[42] Bianco P., Kuznetsov S. A., Riminucci M., Gehron Robey P. (2006). Postnatal skeletal stem cells. *Methods in Enzymology*.

[43] Barry F. P., Murphy J. M. (2004). Mesenchymal stem cells: clinical applications and biological characterization. *The International Journal of Biochemistry & Cell Biology*.

[44] Nuttall M. E., Gimble J. M. (2000). Is there a therapeutic opportunity to either prevent or treat osteopenic disorders by inhibiting marrow adipogenesis?. *Bone*.

[45] Justesen J., Stenderup K., Ebbesen E. N., Mosekilde L., Steiniche T., Kassem M. (2001). Adipocyte tissue volume in bone marrow is increased with aging and in patients with osteoporosis. *Biogerontology*.

[46] Fang S., Li Y., Chen P. (2019). Osteogenic effect of bone marrow mesenchymal stem cell-derived exosomes on steroid-induced osteonecrosis of the femoral head. *Drug Design, Development and Therapy*.

[47] Wu F., Jiao J., Liu F. (2019). Hypermethylation of Frizzled1 is associated with wnt/beta-catenin signaling inactivation in mesenchymal stem cells of patients with steroid-associated osteonecrosis. *Experimental & Molecular Medicine*.

[48] Duque G. (2008). Bone and fat connection in aging bone. *Current Opinion in Rheumatology*.

[49] Liu H. Y., Wu A. T., Tsai C. Y. (2011). The balance between adipogenesis and osteogenesis in bone regeneration by platelet-rich plasma for age-related osteoporosis. *Biomaterials*.

[50] Choi Y. J., Song I., Jin Y. (2017). Transcriptional profiling of human femoral mesenchymal stem cells in osteoporosis and its association with adipogenesis. *Gene*.

