# Supplementary figures and images for: Increased COUP-TFII Expression Mediates the Differentiation Imbalance of Bone Marrow-Derived Mesenchymal Stem Cells in Femoral Head Osteonecrosis
Source: Biomed Res Int. 2019 Dec 8;2019:9262430. doi: 10.1155/2019/9262430 (PMC6925929; doi:10.1155/2019/9262430)

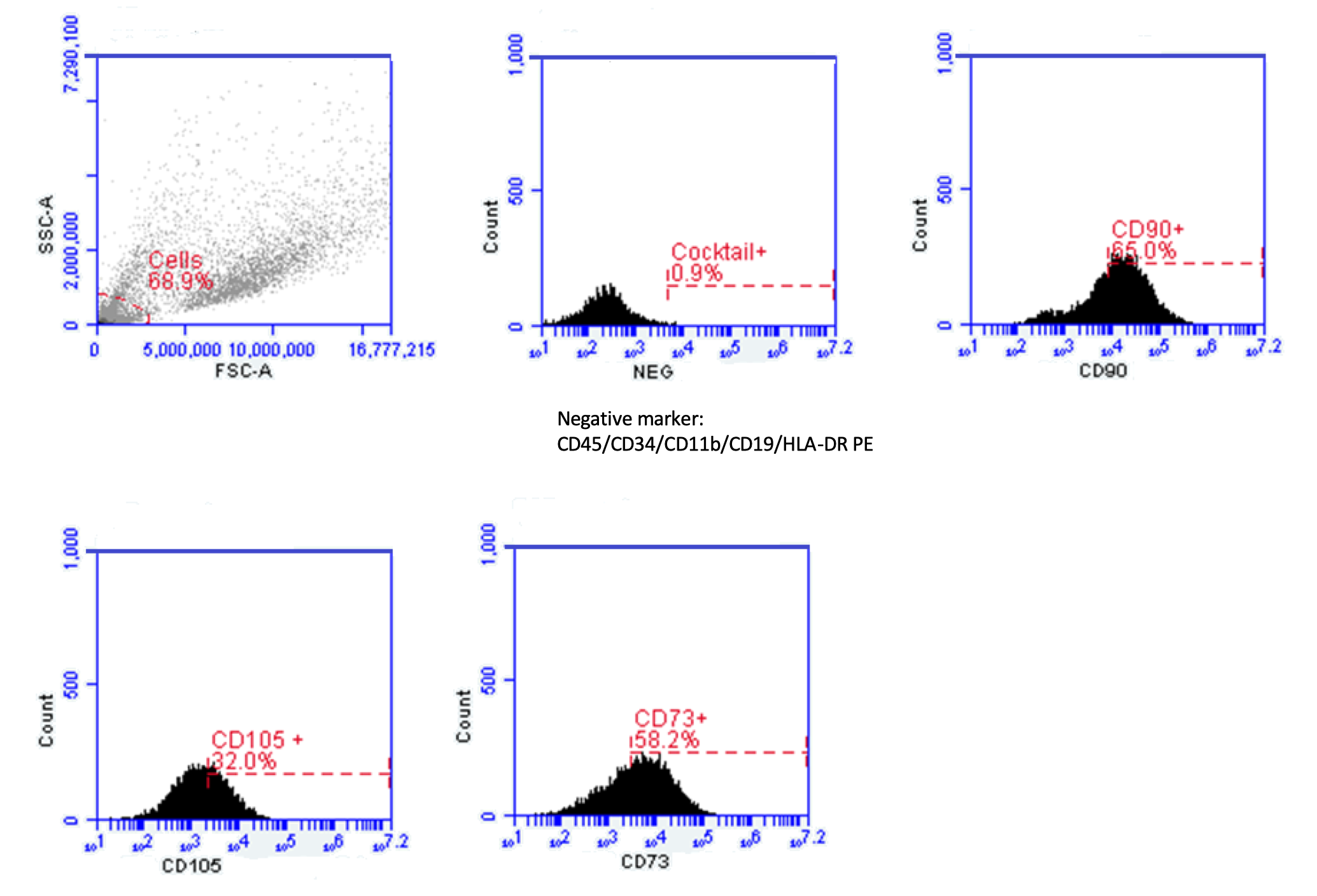

Supplement: Supplementary Materials — Flow cytometric analysis of bone marrow mesenchymal stem cells (BMSCs). After culturing for 16 days, the majority of bone marrow-derived stem cells express the markers CD90, CD105, and CD73, which are characteristic of mesenchymal stem cells, and are negative for CD45/CD34/CD11b/CD19/HLA-DR PE. [file 9262430.f1.png]
